# Distributed manufacturing of an open-source tourniquet testing system

**DOI:** 10.1016/j.ohx.2023.e00442

**Published:** 2023-06-16

**Authors:** Dawei Liu, Apoorv Kulkarni, Victoria F. Jaqua, Christina A. Cole, Joshua M. Pearce

**Affiliations:** a Department of Electrical and Computer Engineering, Western University, London, Canada; b Open Source Medical Supplies, Washington DC, USA; c Ivey Business School, Western University, London, Canada

**Keywords:** Open hardware, Pressure sensor, Sensors, Tourniquet, Frugal engineering, Frugal biomedical

## Abstract

Tourniquets are effective for casualty-prevention in emergency situations. The use of centrally-manufactured commercial tourniquets, however, is not always possible due to supply chain disruptions. The open-source hardware model has been applied to overcome these disruptions in humanitarian crises and several low-cost digitally manufacturable open-source tourniquets have been developed. With the low reliability of improvised tourniquets, it is important to ensure that distributed manufacturing of tourniquets is effective and safe. Tourniquets can be tested, but existing tourniquet testers are expensive, bulky, and complex to operate, which limits their accessibility to an even greater extent than tourniquets in extreme settings. This article fulfills a need by providing a small, transportable, open-source additive-manufactured tourniquet tester that enables inexpensive and accurate testing of tourniquets against known clinical parameters. The <$100 tourniquet tester is validated and tested for operating force of tourniquets in the field or in distributed manufacturing facilities. The tourniquet tester has a significant economic and operational advantage compared to proprietary counterparts available on the market. Once calibrated with a blood pressure monitor, the built-in LCD displays the measuring range of the tester as 0 to 200 N, which is enough to test the validation of all tourniquets.

## Specifications table

**Table:** 

Hardware name	Tourniquet tester
Subject area	Engineering and materials science
Hardware type	Field measurements and sensors
Closest commercial analog	Emergency Tourniquet Trainer – Arm (USD $1,529) https://militarymoulage.com/shop/p/emergency-tourniquet-trainer-arm
Open-source license	*GNU General Public License v.3; CERN OHL v2*
Cost of hardware	USD$65.30 (CAD$89.90)
Source file repository	http://doi.org/10.17605/OSF.IO/DGTW4

## Hardware in context

A tourniquet is a device that applies pressure to a limb to stop bleeding and prevent shock. Tourniquets are widely used in emergency situations, such as combat injuries, mass casualty events, or accidents [1]. Access to tourniquets and associated training continues to develop as a critical first response to penetrating trauma in the civilian population [2], [3]. In 1999, Walter Reed Army Institute of Research collaborated with Oregon State University to evaluate existing tourniquet systems within U.S. military combat medicine [4]. This research, combined with casualty-prevention data from American military operations, resulted in the creation of the Combat Application Tourniquet (CAT) and Joint Theater Trauma Systems (JTTS) [5], [6], which would eventually lead to the Committee on Tactical Combat Casualty Care (CoTCCC) [7], and new prehospital treatment protocols from the American College of Surgeons (ACS) and Advanced Trauma Life Support (ATLS) [8]. By the mid-2000 s, the successful implementation of tourniquets for hemorrhagic control in combat settings was influencing civilian prehospital treatment protocols worldwide [9], [10]. Casualty-reducing protocols from ATLS, ACS, TCCC, and Stop the Bleed all stress field triage, stabilization, and external hemorrhage control with tourniquets as key factors in prehospital survival [11], [12], [13], [14].

**Fig. 1:**
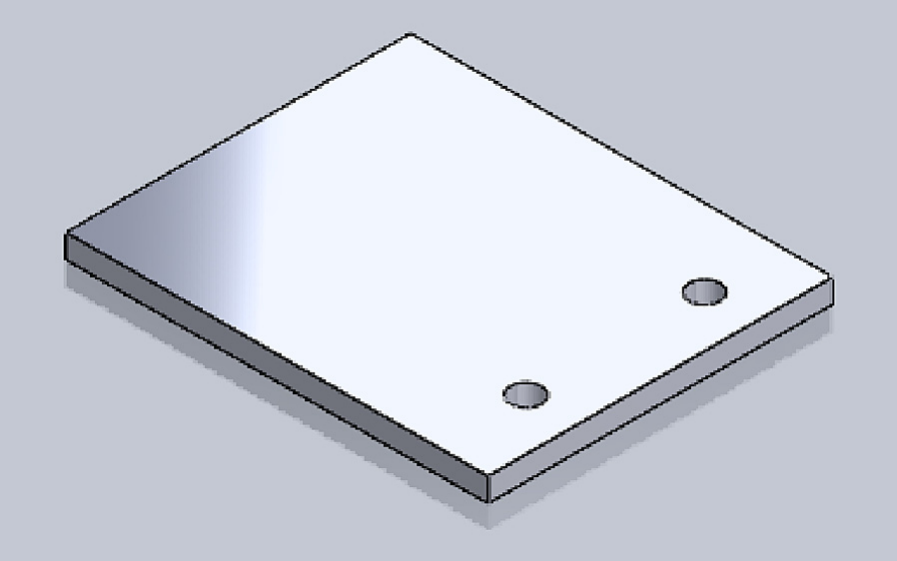
3-D printed Amplifier.

The use of commercial tourniquets, however, is not always possible. For example, in 2014, the United Nations Office for the Coordination of Humanitarian Affairs observed significant civilian casualties during a four-week Gaza War [15], [16]. These fatalities resulted from penetrating gunshot wounds and crush injuries from shelling. Dr. Mohammed Al-Attar observed a CAT tourniquet (brought in by a medical colleague) but, due to the Israeli blockade, it was impossible to afford or import CAT-style tourniquets into Gaza [17], [18]. Al-Attar, the Director of Emergency Operations at the General Directorate of Civil Defence, initiated hemorrhage control and improvised tourniquet training with Gaza’s four ambulance services and the Hayat Center for Emergency & Crisis Management [18], [19], [20]. Although first responders became adept at applying improvised tourniquets, it became apparent that improvised tourniquets had low reliability, were challenging to place effectively over the wound and required extensive assembly time when compared to hemorrhage speed [18]. See (Fig. 1).

Overcoming medical equipment supply chain challenges while maintaining uniformity and thus reliability is often accomplished by utilizing open-source hardware designs [21], [22], [23] coupled with digital manufacturing [24], [25]. In this model, designs are shared with an open-source license and then individuals (do-it-yourself: DIY) [26], [27] or companies/organizations working together (do-it-together: DIT) [28], [29], [30] are free to download and replicate them. The opportunity to overcome supply disruptions in humanitarian crises [31], was recently brought into sharp focus during the COVID-19 pandemic [32], [33], [34], [35], [36], [37], [38], [39]. There is hope that such an approach can be used to solve less acute shortages of medical equipment ranging from tools to help those suffering from chronic respiratory diseases [40] to the needs of surgeons [41]. A large portion of the promise of this approach is the ability to radically reduce costs as enormous economic values are created with replication from electronics [42], scientific tools [43], [44], [45], [46] and even MRI machines [47]. With reduced capital costs, which are generally around 90% [48], and value creation through distributed replication [49], the return on investment for designing new high-value products in the science and medical field is high (hundreds to thousands of percent) [50].

**Fig. 2:**
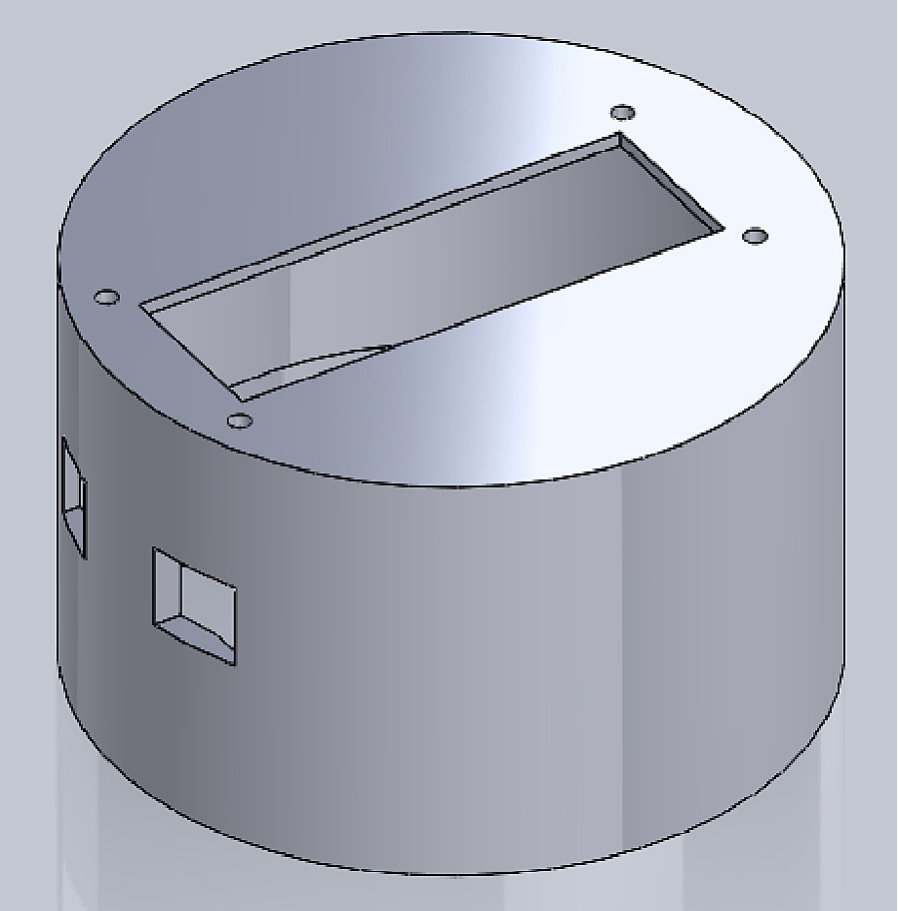
3-D printed Cap.

Thus, this open-source 3-D printing approach was used following the success of the Glia stethoscope 3-D printed in Gaza [51]. Al-Attar partnered with Glia’s Gaza-based engineers to create a 3-D printed tourniquet that matched the clinical effectiveness of a CAT and published it on GitHub in 2017 [52]. On March 31, 2018, the first four hours of the Great March of Return resulted in 3,500 Palestinian casualties, roughly half of which were gunshot wounds [53], [54]. The Glia Gaza team immediately initiated tourniquet design improvements based on field training and feedback from Gaza EMS, often under live fire, and ramped production from 60 to 100 units/week to 200 units/week throughout 2018 and 2019 [18], [55], [56]. The tourniquets were effective. Early tourniquet field application, combined with Trauma Stabilization Points managed by the World Health Organization and Gaza EMS, was a critical factor in a 0.03% fatality rate among 5,969 gunshot victims [57]. This high number of casualties requiring tourniquets gave Glia the opportunity to iterate design changes, perform bench tests, and deploy bench-cleared units directly into the field [58]. See (Fig. 2).

**Fig. 3:**
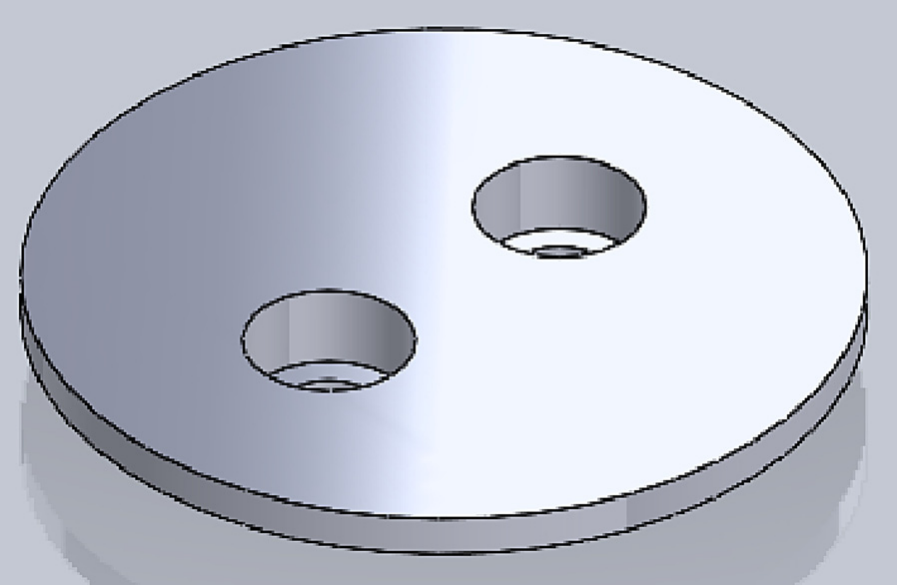
3-D printed Flat.

In March 2022, Glia announced the tourniquet design for open-source emergency use in Ukraine and formed a partnership with Open Source Medical Supplies (OSMS) to coordinate decentralized additive manufacturers [59], [60]. Glia and OSMS quickly saw that additive manufacturers were changing the design to make it easier to print and departing from clinical efficacy [61]. Additionally, generic CAT clones and independently designed tourniquets flooded the Ukraine market [62]. Dr. Tarek Loubani, Glia Medical Director, visited Ukraine in May 2022 and observed a broad spectrum of tourniquet unit testing procedures [63]. The wide range of qualities for donated tourniquets, clones, and altered 3-D printed tourniquets has created a severe need for a tourniquet tester. There are no publicly accessible standards for unit testing a tourniquet, either for end-users or tourniquet manufacturers, which has created confusion in validating tourniquet clinical field performance. ASTM has been developing test fixture and tourniquet testing standards since 2016, but these standards are not yet publicly available [64]. Effective tourniquet application requires a combination of end-user training and mechanically effective tourniquet unit performance. The end-user must be confident a tourniquet dependably meets a clinical standard before patient application [65]. Applying a tourniquet incorrectly can cause complications, such as nerve damage, tissue ischemia, or amputation. Therefore, it is important to ensure that tourniquets are effective and safe before using them on patients. See (Fig. 3).

Tourniquet performance can be validated by a tourniquet tester, a device which measures the pressure applied by the tourniquet and the blood flow in the limb [66], [67]. Tourniquet testers can help evaluate the quality of different types of tourniquets, such as pneumatic, elastic, or mechanical systems [68]. They can also help train medical personnel on how to use tourniquets properly and monitor their effects [69], [70]. Unfortunately, existing tourniquet testers are expensive, bulky, or complex to operate (see Table 1). They may also require specialized equipment or calibration procedures that limit their accessibility and usability in low-resource settings [71]. There is a clear need for an open-source additive manufactured unit tester that would allow a manufacturer or end-user to inexpensively and accurately test their device against a known clinical parameter, an important resource for conflict-affected and resource-limited communities.

**Table 1:** Commercial tourniquet test systems.

**Product**	**Cost**	**Functions**
TrueClot Tourniquet Trainer [72]	USD$725	This device has interior blood flow. When the tourniquet is correctly applied, hemostasis is achieved on simulated blood flowing from the wound. Cannot show data.
Tourniquet Task Trainer Arm [73]	USD$490	This device has interior blood flow. When the tourniquet is correctly applied, hemostasis is achieved on simulated blood flowing from the wound. Cannot show data.
Chi Systems’ HapMed Tourniquet Trainer [74]	Not available	This device has LEDs and screens, which can demonstrate data and hemostasis indicators.
Sim Limb Bleed Control Tourniquet Trainer [75]	USD$365	This device simulates blood flow from a wound. Cannot show data.
Emergency Tourniquet Trainer – Arm [76]	USD$1529	Simulated upper arm with realistic wounds connected to a pump. When the tourniquet is correctly applied, hemostasis is achieved on simulated blood flowing from the wound.
Humimic Tourniquet Arm Trainer [77]	USD$500	Simulated upper arm with realistic wounds and interior blood flow. When the tourniquet is correctly applied, hemostasis is achieved on simulated blood flowing from the wound. Does not show data.
Humimic Tourniquet & Wound Packing Leg Trainer [78]	USD$632	Simulated upper leg with realistic wounds and interior blood flow. When the tourniquet is correctly applied, hemostasis is achieved on simulated blood flowing from the wound. Does not show data.
3B Scientific Hemorrhage Control Arm Trainer P102 [79]	USD $1,975	Simulated upper arm and shoulder with realistic wounds and interior blood flow. When the tourniquet is correctly applied, hemostasis is achieved on simulated blood flowing from the wound. Does not show data.

**Fig. 4:**
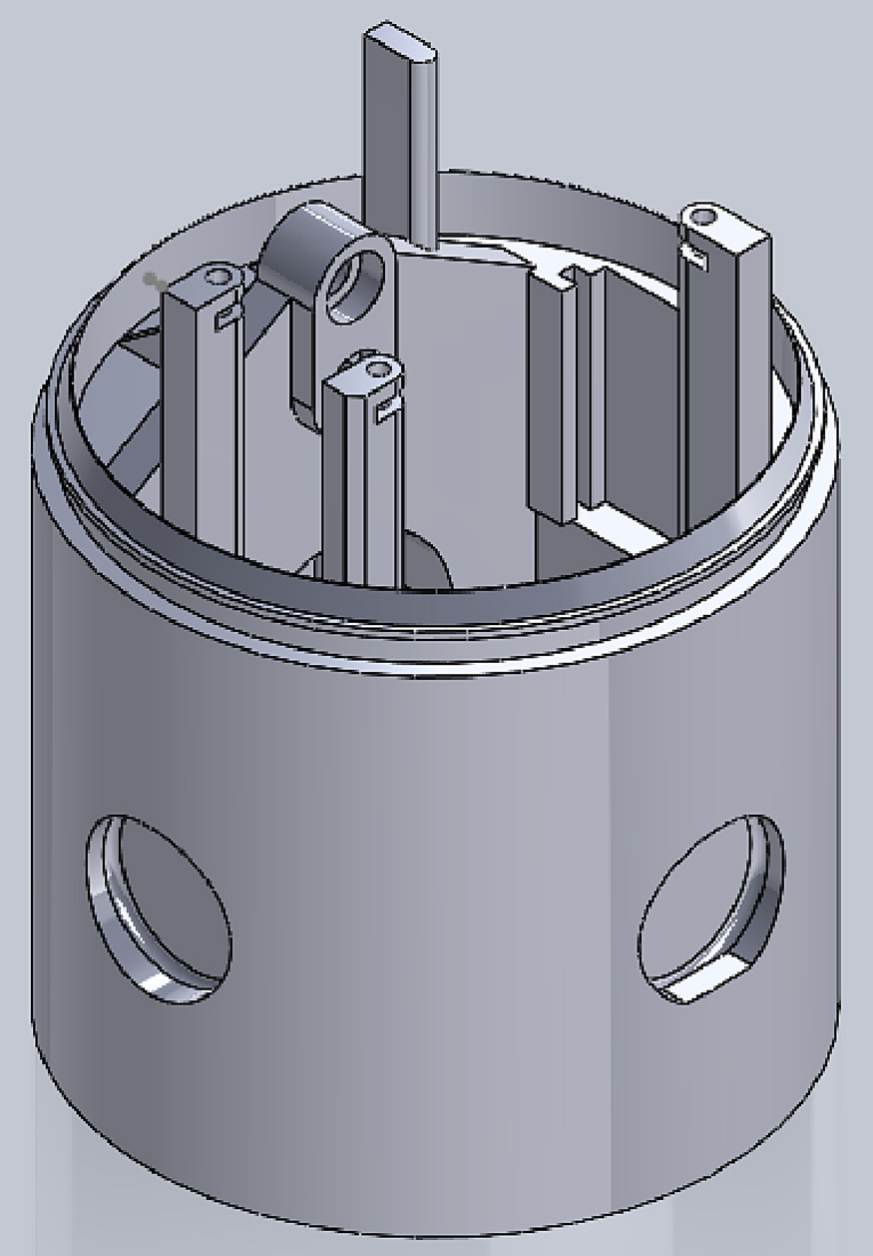
3-D printed thigh inside cylinder.

To fill this need, this article describes an open-source 3-D printed tourniquet tester that is low-cost, portable, and easy to both manufacture and deploy across diverse scenarios. The design and fabrication process of the device using common materials and tools is described. The tourniquet tester is validated for functionality and accuracy by comparing it with a commercial tourniquet tester on different types of tourniquets. A low-cost calibration process is demonstrated, and potential errors are quantified. The advantages and limitations of the device are discussed and future work is outlined. See (Fig. 4).

## Hardware description

An open-source 3-D printable tourniquet tester is developed. All the custom mechanical components can be fabricated with a low-cost desktop fused filament fabrication-based RepRap-class 3-D printer. The electronic components are all open source and readily available off-the-shelf from a wide variety of vendors. The compact design of the tourniquet tester makes it easy to manufacture and easy to transport. The electronic circuit diagrams are provided to power the testing unit via a USB input. The tourniquet tester is validated and tested on Glia units as well as CAT units for testing the operating force of tourniquets in the field or in distributed manufacturing facilities (e.g., fab labs, makerspaces, 3-D print shops, libraries, schools, or in volunteers’ homes). As of this writing, there is no agreement on testing standards from the tourniquet manufacturers, so this open-source 3-D printed tourniquet tester provides a means of democratizing the environments which require tourniquet use. The tourniquet tester has a significant economic and operational advantage compared to proprietary counterparts available on the market.

• Designed for distributed manufacturing settings and resource-limited communities seeking to validate donated commercial tourniquets, validate novel/generic commercial tourniquets, and those made with distributed manufacturing consistent with the Glia and other types of open-source tourniquets.
• Low production cost: All the housing components are manufactured by 3-D printing and assembled easily and quickly.
• Portable and durable: The compact design makes the testing unit easy to transport and can be made from the choice of high-durability 3-D printing materials.
• Simple operation: Once the tester is calibrated with a blood pressure cuff, it does not need any extra steps or adjustments. The pressure reading is directly shown on the LCD display. According to the measuring range of the load cell (0–20 kg), the measuring range of the tester is 0 to 200 N and 0 to 698.2 mmHg (depending on the calibration factor S), which is enough to test the validation of the tourniquets.

## Design files summary

The design files are summarized in Table 2.

**Table 2:** Design files.

**Design file name**	**File type**	**Open source license**	**Location of the file**
Amplifier mounting	STL&STEP	*CERN OHL v2*	https://osf.io/hf4dm
Cap	STL&STEP	*CERN OHL v2*	https://osf.io/dajfv
Flat	STL&STEP	*CERN OHL v2*	https://osf.io/gwbtn
thigh inside cylinder	STL&STEP	*CERN OHL v2*	https://osf.io/vqwc9
Tournbutton – M	STL&STEP	*CERN OHL v2*	https://osf.io/gvmkr
Tournbutton-T	STL&STEP	*CERN OHL v2*	https://osf.io/a6xc8
Unit tester	ino	*GNU General Public License v.3*	https://osf.io/zr79k

**Fig. 5:**
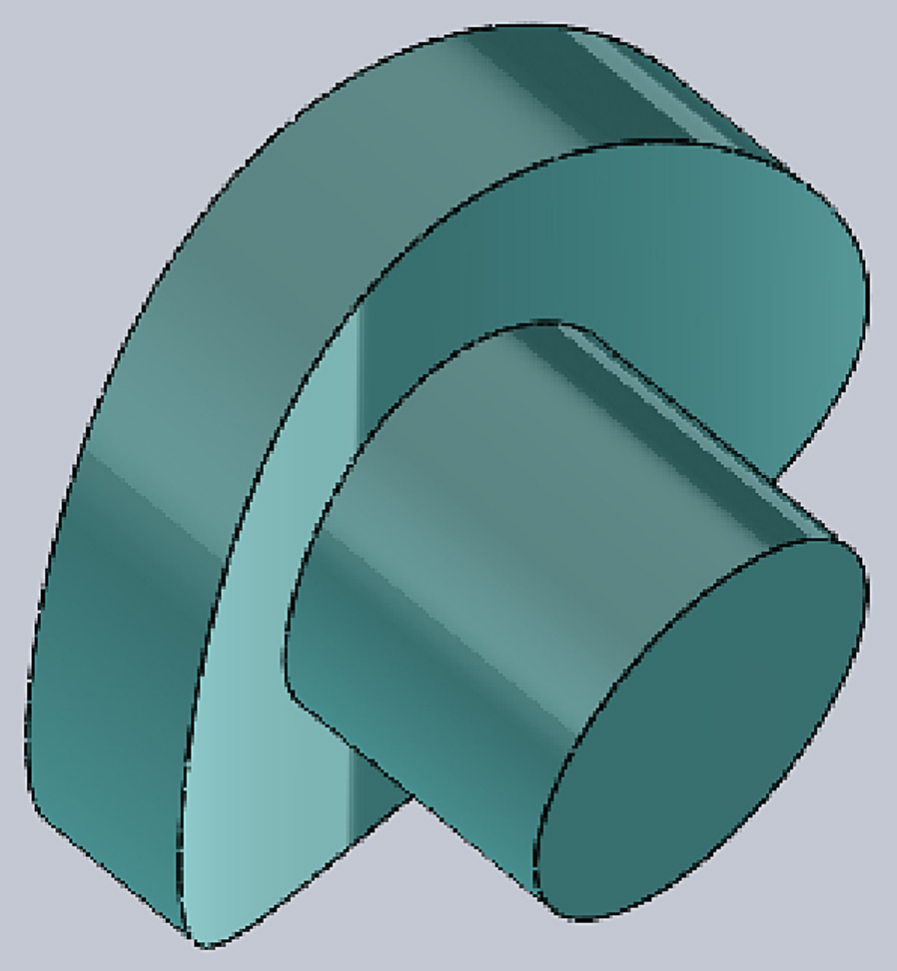
3-D printed Tournbutton-M.

The tourniquet tester is cylindrical in shape and has an LCD screen at the top to display the measured values. The housing of the tester is made of hard thermoplastic material, and there are four assembly holes for round buttons made of flexible thermoplastic polyurethane (TPU) material. The tester housing is 3-D printed and can be customized if necessary, which means that different sizes of housings can be made to suit different test conditions. See (Fig. 5).

File 1. Amplifier mounting: This component is part of the amplifier assembling. To hold the amplifier in place, M2.5 bolts and nuts are used to secure the amplifier HX711 to the Amplifier mounting and then inserted the part into the notches reserved in the cylinder.

File 2. Cap: top part of the housing, LCD is mounted on it. There are two holes on the side for the power supply.

File 3. Flat: disk-shaped part for the load cell. It is the direct interface with Tournbutton-M.

File 4. Thigh inside cylinder: the main part of the housing. Most electronic parts should be mounted on this part.

File 5. Tournbutton-M: the button is made from hard material, for measuring. The cylinder part should be long enough so that it has enough space to be pushed down.

**Fig. 6:**
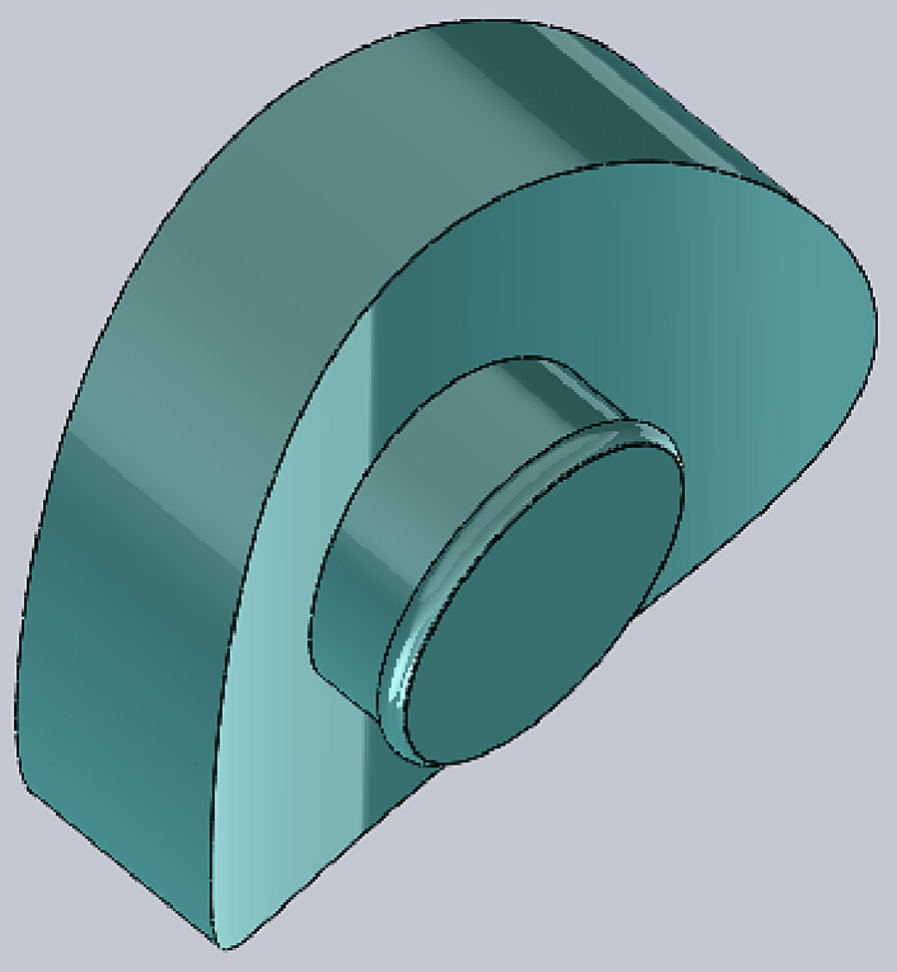
Tournbutton-T.

Tournbutton-T: button made of TPU material, mounted on the inside of the cylinder. It should be thick and flexible enough so that the tourniquet has enough space to generate pressure. See (Fig. 6).

## Bill of materials summary

The full bill of materials can be downloaded from the Open Science Framework [80] containing links to suppliers but is summarized in Table 3. All costs are in Canadian dollars.

**Table 3:** Bill of materials.

**Designator**	**Component**	**Number**	**Cost per/ $CAD**	**Total cost –** **$CAD**	**Source of materials**	**Material type**
1	Thigh inside cylinder	1	0.03/gram	5.1	Amazon	Polymer
2	Cap	1	0.03/gram	1.7	Amazon	Polymer
3	Flat	1	0.03/gram	0.1	Amazon	Polymer
4	Amplifier mounting	1	0.03/gram	0.1	Amazon	Polymer
5	Tournbutton – M	1	0.03/gram	0.3	Amazon	Polymer
6	Tournbutton-T	3	0.03/gram	1.2	Amazon	Polymer
7	20 KG-load cell	1	11.5	11.5	Amazon	Aluminum
8	HX711 amplifier	1	4.5	4.5	Amazon	Integrated circuit
9	Arduino Uno	1	47	47	Amazon	Development board
10	16x2 LCD	1	15	15	Amazon	Module
11	Jump wires	8	0.25	2	Amazon	Wire
12	M2.5*5	3	0.1	0.3	Amazon	Metal
13	M2.5*8	2	0.1	0.2	Amazon	Metal
14	M2.5 nuts	5	0.1	0.5	Amazon	Metal
15	M3	2	0.1	0.2	Amazon	Metal
16	M4	2	0.1	0.2	Amazon	Metal
Total				89.9		

## Build instructions

First, acquire all of the components in the BOM shown in Table 3. For the 3-D printed components detailed in Table 2, Table 3-D print using the setting summarized in Table 4.

**Table 4:** 3-D printing parameters.

**Parameters**	**Other 3-D printed part**	**Tournbutton-T**
Printer	LulzBot TAZ workhorse	Prusa i3 MK3S+
Slicing software	Cura	PrusaSlicer
Materials	PLA (1.75 mm)	TPU (NinjaFlex 1.75 mm)
Nozzle temperature	210 ℃	238 ℃
Printing bed temperature	60 ℃	50 ℃
Wall thickness	2 mm	
Speed	60 mm/s	60 mm/s
Infill	20% (Grid)	10%-15% (Gyroid)
Layer height	0.38 mm	0.2 mm
Support	No	No

## Assembling the electronic components

**Fig. 7:**
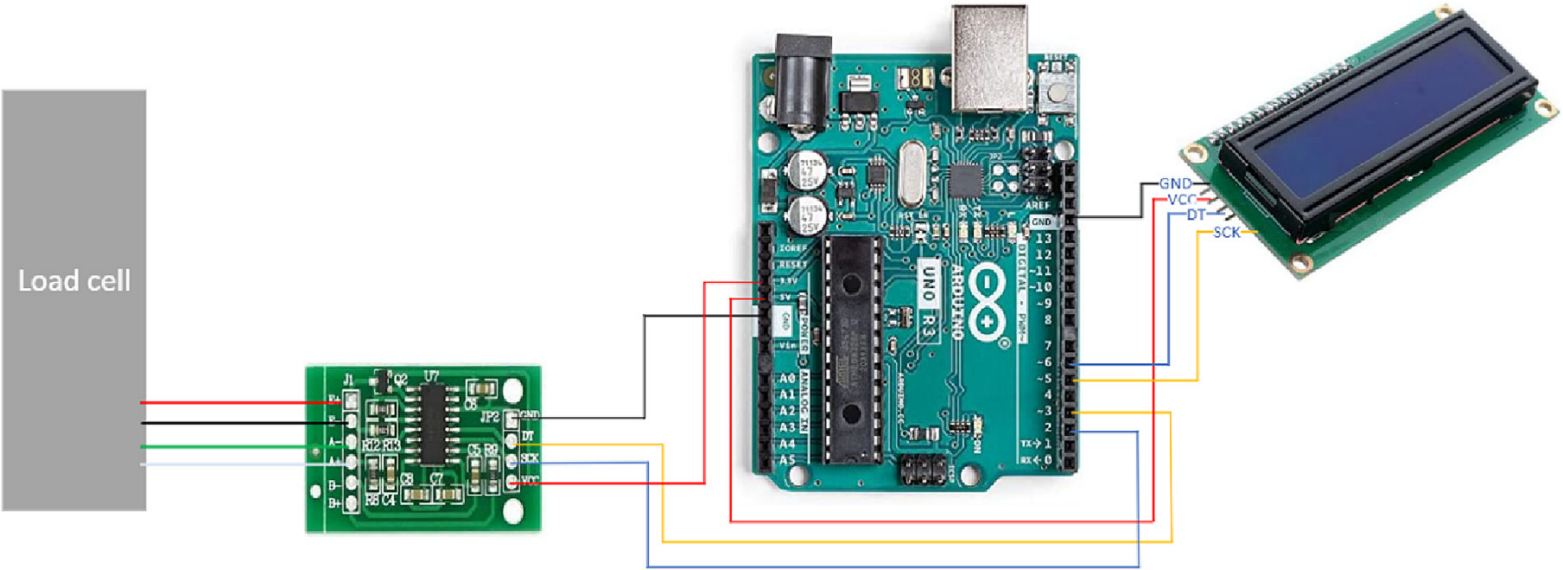
Wire connections.

Connect the HX711 amplifier to the load cell. For more reliability, solder the wires on the load cell to the amplifier. Then, use the jump wires to connect the LCD and amplifier to the Arduino Uno board as shown in Fig. 7. The wiring is further detailed in Table 5.

**Table 5:** Wire connections.

**Load cell to amplifier**	**Amplifier to Arduino:**	**LCD to Arduino:**
Red → E+	VCC → 5 V	VCC → 3.3 V
Black → E-	GND → GND	GND → GND
Green → A+	CLK → pin 2	DT → pin 6
White → A-	DAT → pin 3	SCK → pin 5

## Build the unit tester housing.

**Fig. 8:**
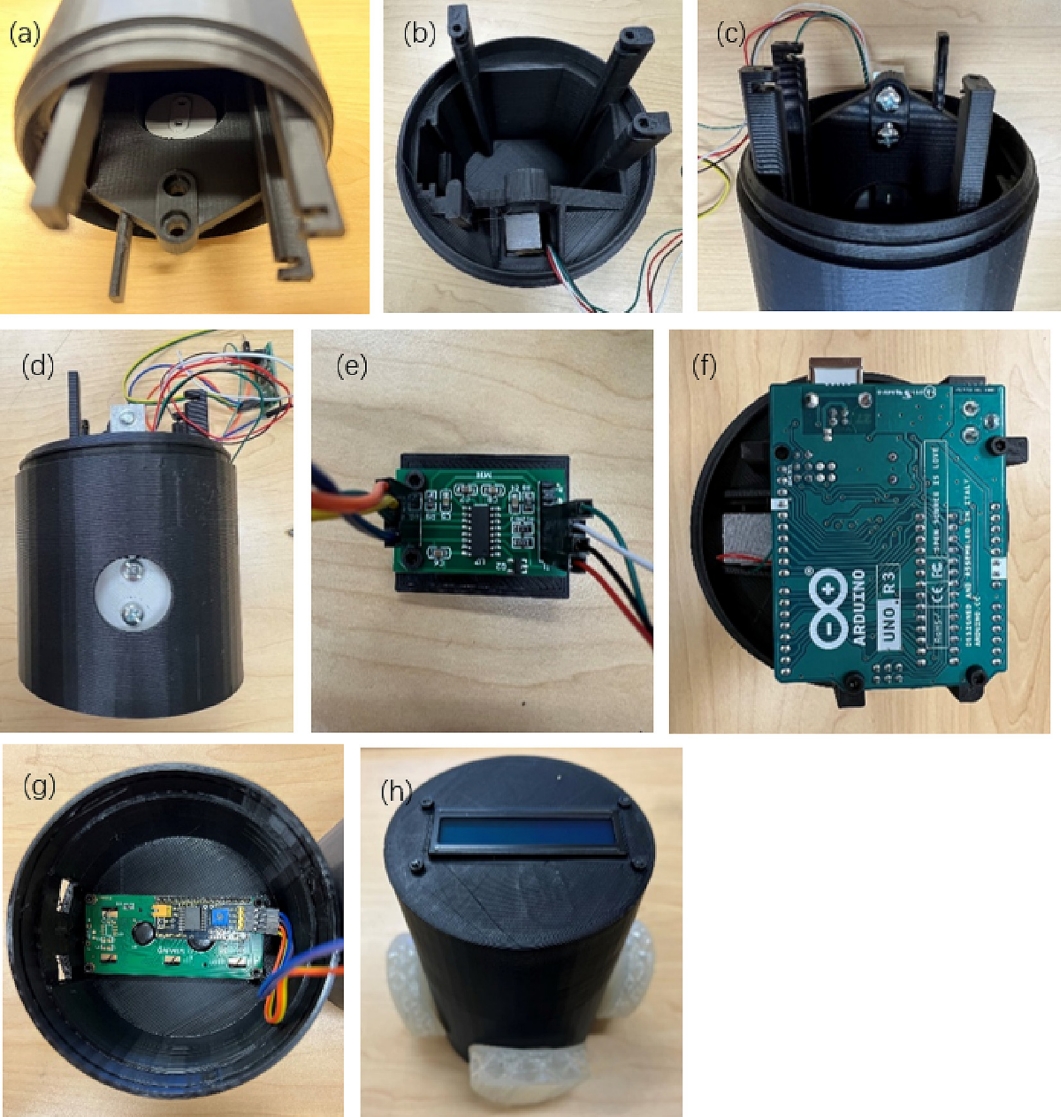
Assembly steps of physical components. (a) Put the flat part into the groove, (b) Insert the load cell, (c) Fix the load cell, (d) Fix the flat, (e) Fix the amplifier, (f) Fix the Arduino U no board, (g) Fix the LCD on the cap, and (h) Insert the TPU button.

Once all the design files are 3-D printed, the assembly process is as follows with the steps shown in Fig. 8.

Put the flat into the groove inside the cylinder.

2. Insert the load cell into the reserved hole.

3. Use M4 bolts to fix the load cell on the fixing point.

4. Use M3.5 bolts to fix the flat on the load cell.

5. Use M2.5 bolts and nuts to fix the amplifier on the 3-D printed amplifier part, and then insert the part into the reserved notch inside the cylinder.

6. Use M2.5 bolts and nuts to fix the Arduino U no board to the four posts on the cylinder. Only three of them have holes for fixing and one is for support only.

7. Use M2.5 bolts and nuts to fix the LCD on the cap, and then cover the cap, with the holes reserved on the cap aligned with the power and USB slots of the Arduino board.

8. Insert the TPU button into the reserved hole on the cylinder.

The assembly is complete.

Because of the use of 3-D printing technology, the design supports changing the size to suit different situations. The cap and the cylinder are connected using a snap fit, which makes installation easy and saves mounting materials such as bolts and nuts.

3. To assemble the calibration platform (optional). See BOM on the OSF [80].

1. Using M3 bolts to mount the linear rail fixing part to the board.

2. Attach the linear rail to the fixing part.

3. Attach the 3-D printed platform to the linear block.

The calibration platform makes the calibration of the tester easy but is not necessary.

## Operation instructions

Before testing the tourniquet, calibration is required. Due to the nature of the sensor, the calibration consists of two parts,

• Calibration of the load cell (Force reading)
• Calibration of the tourniquet tester unit as a whole (Pressure reading)

### Calibration of the force measurement

**Fig. 9:**
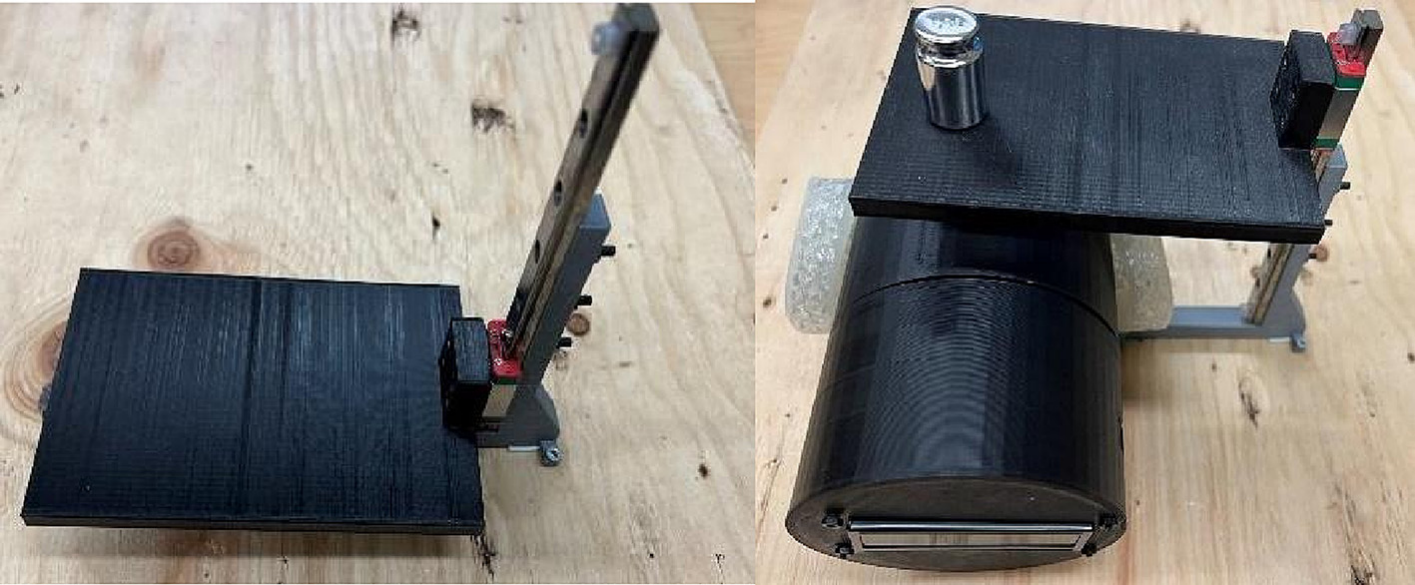
Optional calibration platform shown empty on the left and calibrating the tester on the right.

Initially, the load cell can be calibrated using known weights. Known weights can be placed on the tournbutton-M while the tester is placed flat. This method has limitations on the weight that can be applied. Alternatively, a calibration device may be employed, which consists of a wooden plate, a linear rail, bolts and nuts, and other 3-D printed parts as Fig. 9 shows. Once assembled, the tester is placed beneath the 3-D printed platform and connected to a computer. By manipulating the weights on the platform, data can be obtained.

To get the calibration factor of the force measurement, an open-source calibration code [80] is utilized to acquire this factor. To apply this calibration code, first, it needs to be uploaded to the Arduino board through Arduino IDE software. There are instructions to be followed in the code including [81]:

1: Set up the tester and start the sketch without a weight on the tester.
2: Once readings are displayed place the weight on the scale.
3: Press +/- or a/z to adjust the calibration_factor until the output readings match the known weight.
4: Use this calibration_factor on the unit tester sketch.

**Fig. 10:**
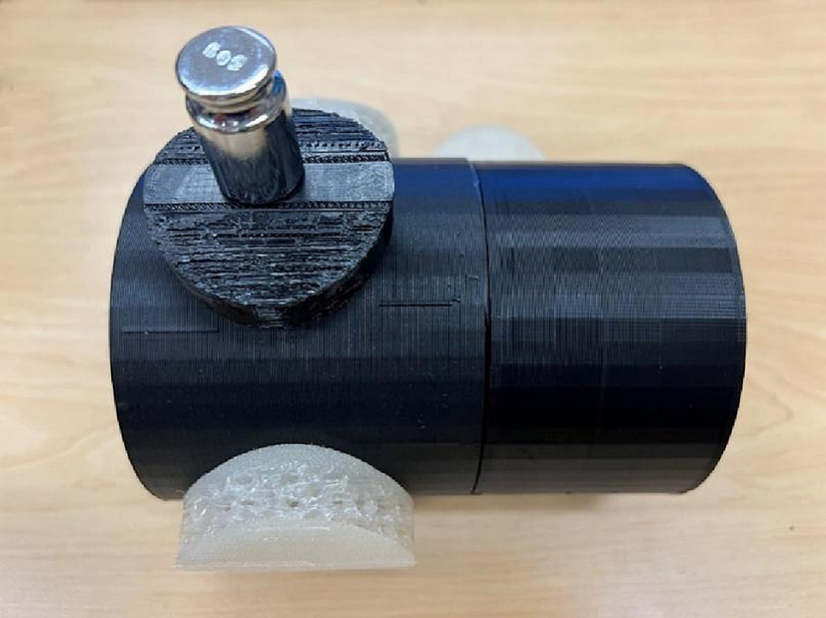
Calibration process with no platform.

**Fig. 11:**
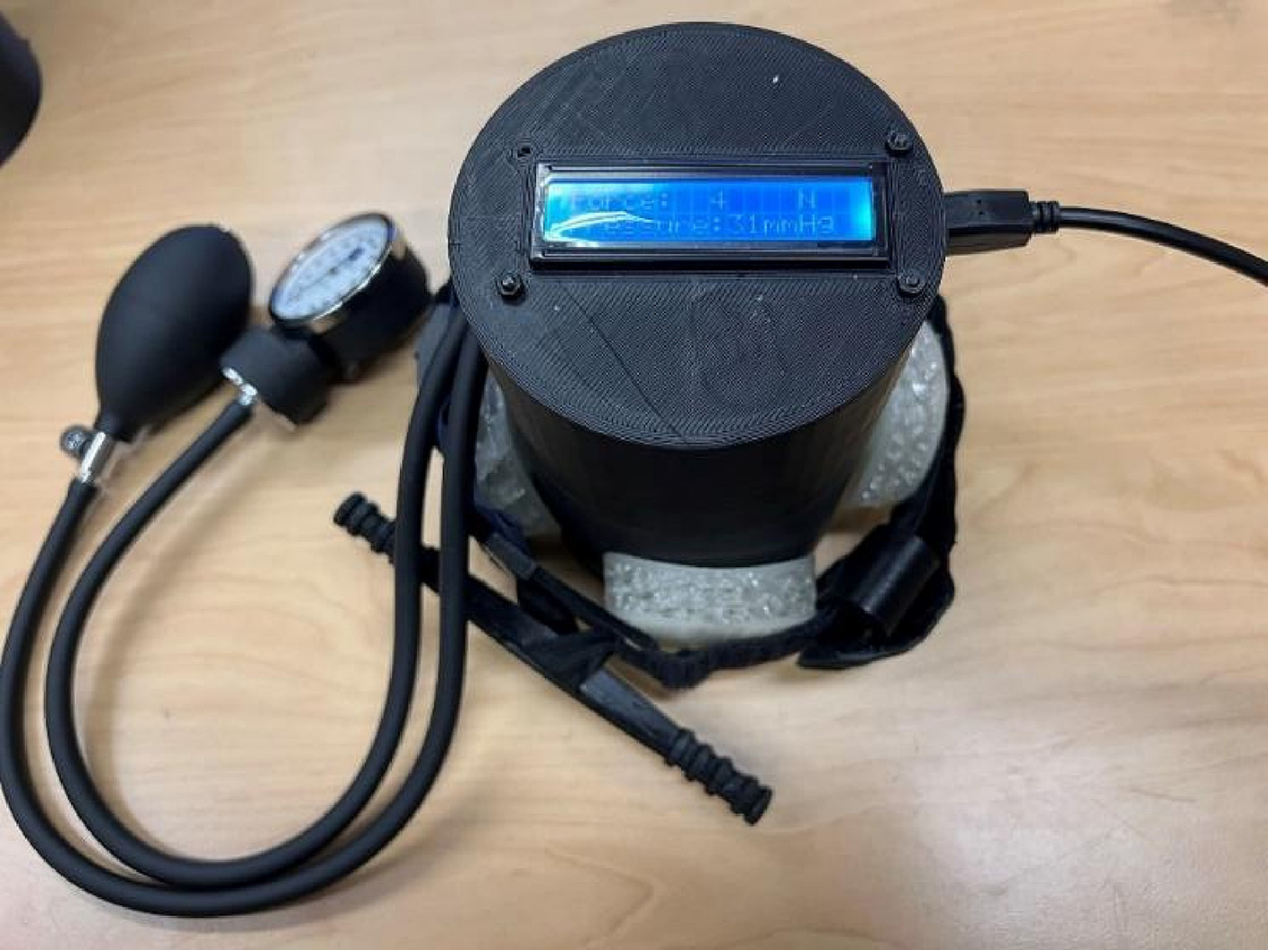
Calibration against the blood pressure cuff.

The example code assumes pounds (lbs). In this project, 100 g was used as the unit. Unit can be changed in the Serial.print(“ lbs”); line to 100 g. The parameter that can be obtained could be a positive or negative number, it depends on the way of implementing the load cell. The factor typically ranges between 1500 and 15000. It should be noted that this parameter may differ depending on the environment in which the load cell is installed, thus necessitating recalibration each time the load cell is reinstalled. See (Fig. 10 and Fig. 11).

### Calibrate the pressure parameter

In most medical and clinical settings, the pressure generated by the tourniquet is recorded in mmHg. As the sensor in the unit tester is a load cell, it can only measure the force being applied, not the pressure. Therefore, the force measurement is converted into pressure, following:

(1) P=F/S[Pascal]

where P is pressure (pascal), F is force (N) and S is surface area (m^3^). Equation (1) requires an area to convert force into pressure, which in this case is the effective surface area acting on the load cell. Various factors such as the force concentration, width of the tourniquet, the number of turns of the windlass, and the part of the tourniquet that touches the button can also influence the readings. To address this issue and obtain more reliable pressure measurements, the method used to measure occlusion pressure was adopted, which uses a manual blood pressure cuff (manual sphygmomanometer) as the calibrating unit [68], [71]. These blood pressure cuffs are widely accessible globally. This approach is divided into two parts. First, determine the optimum position of the blood pressure cuff with reference to the load cell/ “tournbutton-M” part. Second, determining the effective surface area parameter.

**Fig. 12:**
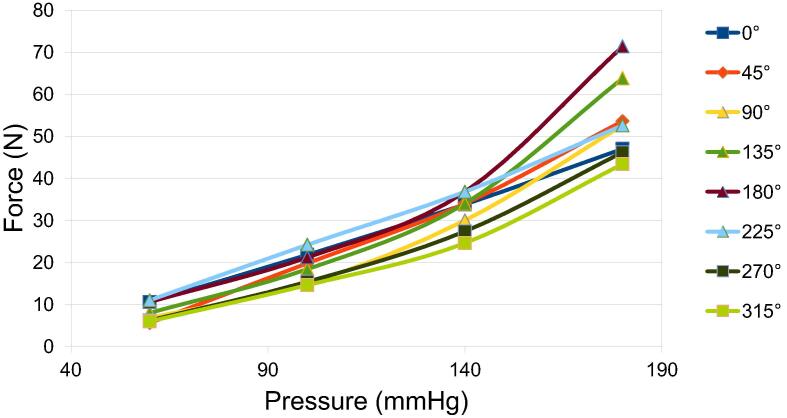
Experiment data of the force measured as a function of the pressure in the various angles of rotation.

**Fig. 13:**
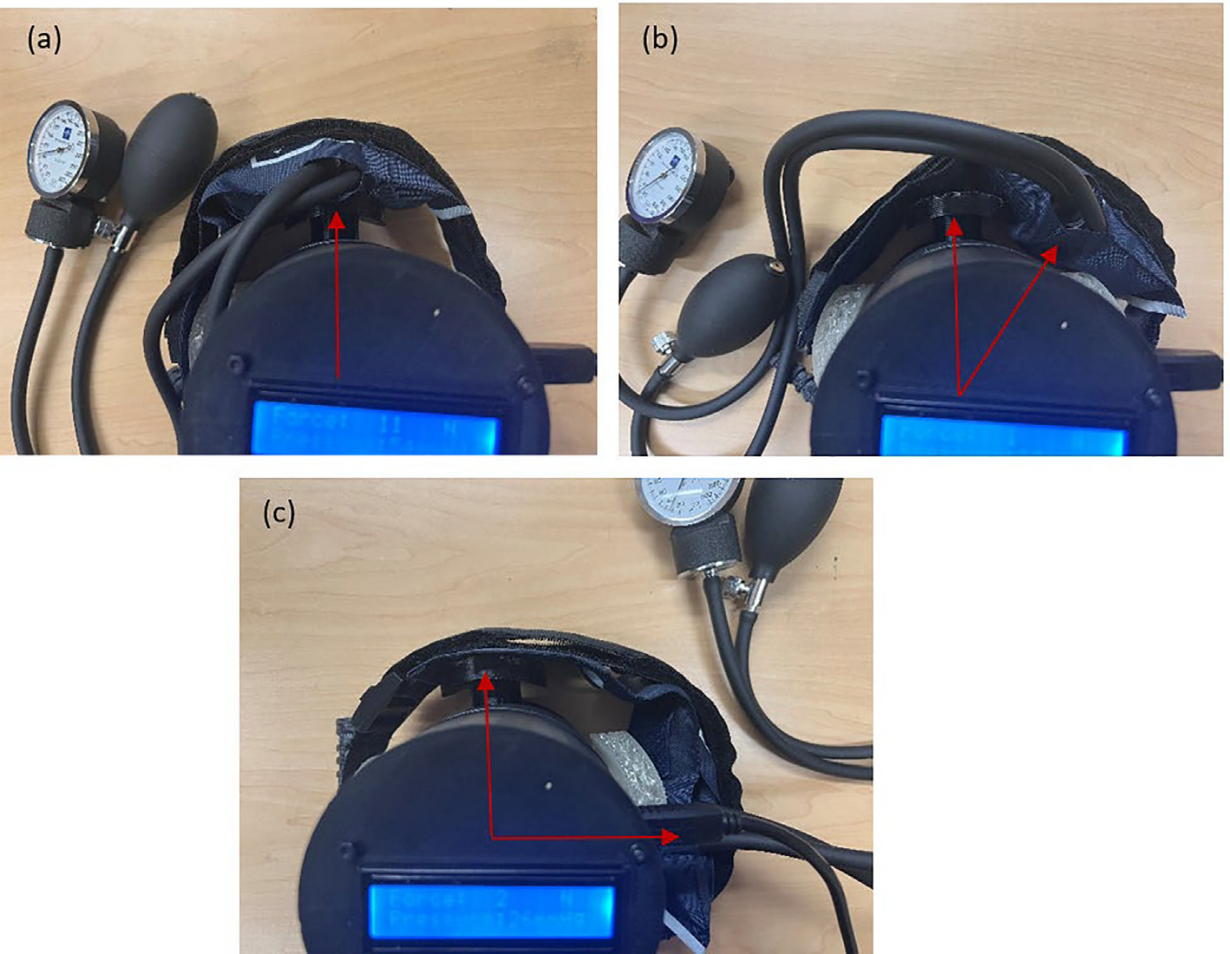
Cuff position at (a) 0 degrees,(b) 45 degrees and (c)90 degrees.

To determine the optimum position of the blood pressure cuff for further testing, a manual pneumatic blood pressure cuff was wrapped around the unit tester, and the tourniquet was applied to the cuff. The pressure generated by the tourniquet was determined by subtracting the original reading from the reading after applying the tourniquet. An infant-sized blood pressure cuff was used to ensure consistency in the measurement position, and five readings were taken at 60 mmHg, 100 mmHg, 140 mmHg, and 180 mmHg, respectively. The cuff was adjusted such that the pneumatic pipe aligns exactly at the center of the tournbutton-M at 0 degrees. The cuff was then rotated by 45 degrees, and the readings were recorded again. This was repeated 8 times from 0 degrees to 315 degrees. Fig. 12 shows the readings of pressure applied by the blood pressure cuff and the force recorded by the load cell of the tourniquet tester as the system was rotated (Fig. 13). The data in Fig. 12 shows similar trends for all the angles of rotation tested. To determine the fixed position of the blood pressure cuff for further testing and validation, the 0-degree angle is chosen, where the cuff is exactly on top of tournbutton-M. This was done because the readings showed a linear relationship between the pressure applied by the tourniquet and the force measured by the load cell at the particular position.

Now, to find out the surface area the procedure is as follows:

1. Inflate the pneumatic blood pressure cuff to about 20 mmHg. Place the blood pressure cuff around the tester in the 0-degree position.

2. Record the force readings at 10 mmHg increments while increasing the pressure from turning the windlass of the tourniquet.

3. The following equations are used to calculate the value of the surface area, S. First the pressure, P is determined:

(2) P=C-20[mmHg]

(3) $$S=F\;/\;\left(133.32*P\right)\;\left[m^{3}\right]$$

where c represents the reading on blood pressure cuff in mmHg, F represents the reading of force (N) on the tester. In equation (2), the initially applied pressure of 20 mmHg is subtracted from the pressure reading. The constant 133.32 is the conversion factor for converting the pressure from mmHg to Pascals.

Measure the force readings at least three times to obtain stable values of force against the applied pressure. Calculate the average of the force measurements and further calculate the surface area parameter S for each of the pressure readings. Finally, the average of all the S values is considered the final calibration factor/surface area parameter. See (Table 6).

**Table 6:** Calibration data.

**Blcuff**	**P**	**F (Average)**	**Surface Area Parameter**
68	48	13.23	0.00207
80	60	17.47	0.00218
90	70	20.53	0.00220
100	80	22.97	0.00215
110	90	26.27	0.00219
120	100	29.50	0.00221
130	110	33.20	0.00226
140	120	36.70	0.00229
150	130	40.27	0.00232
160	140	44.17	0.00237
170	150	47.60	0.00238
180	160	51.20	0.00240
190	170	55.20	0.00244
200	180	58.33	0.00243
210	190	61.87	0.00244
220	200	65.93	0.00247
230	210	70.60	0.00252
240	220	73.50	0.00251
250	230	77.23	0.00252
		**Average**	**0.00233**

Put S in the equation in the code “unit tester” [80].

**Fig. 14:**
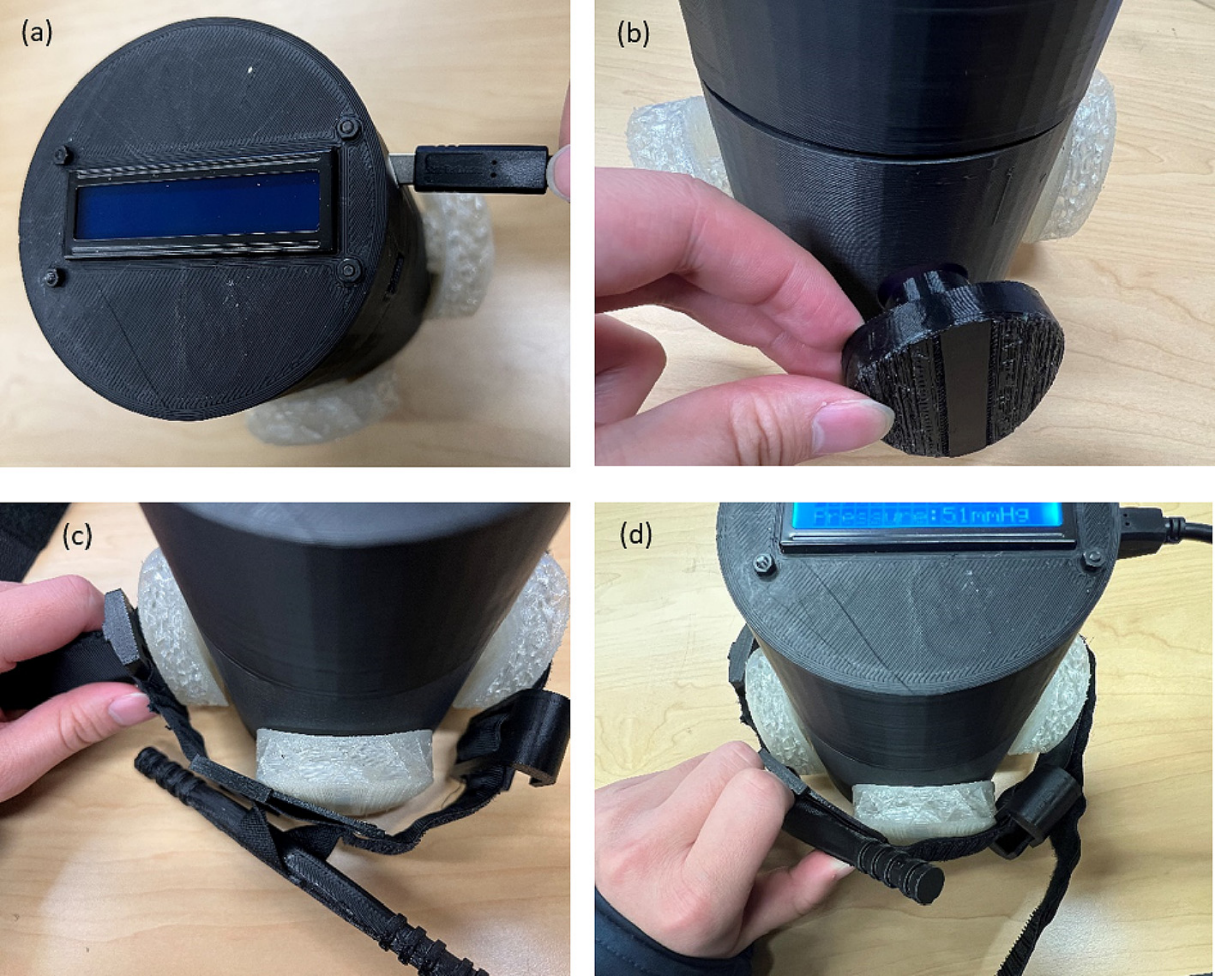
Measurement process (a) Plug in the power cord (b) Insert Tournbutton-M (c) Wrap the tourniquet to the tester (d) Turn the tourniquet windlass.

Once calibration is complete, the tester is ready for measurement. The measurement process is shown in Fig. 14 and is as follows:

1. Plug in the power cord and the tester starts up.

2. Insert Tournbutton-M into the hole used for measurement.

3. Wrap the tourniquet around the tester, and make sure that the tourniquet is pressing the Tournbutton-M, and the Tournbutton-M stands vertically on the flat (for the force can distribute evenly).

4. Turn the tourniquet windlass and the corresponding value will appear on LCD.

## Validation

To validate the calibration procedure,

1: Inflate air into the blood pressure cuff to about 20 mmHg.
2: Put the blood pressure cuff in between the tourniquet and the tester.
3: Test the tourniquet on the unit tester. Record the pressure on the tester and blood pressure cuff.
4: Compare the difference between these data.

If the readings do not have a large gap (standard), then it can be concluded that the unit tester is valid.

Fig. 8 shows the validation data, where the plot is an average of 5 tests of blood pressure cuff readings vs the calibrated tourniquet tester readings. The results fit within a relatively straight line with the R^2^ value of 0.99. The slope of the line is 1.07 to confirm that the blood pressure cuff measurements and the tourniquet measurements correspond to the same value. Thus, it can be confirmed that the tester unit is validated. The same procedure can be used in any distributed manufacturing facility making the testers. The testers can then be deployed to tourniquet manufacturers.

To demonstrate how open-source tourniquet testers could perform across a diverse ecosystem, five tourniquets of varying style from geographically-dispersed manufacturers were tested, including those from volunteer fabricators.See Appendix A1 for photographs of various tourniquet types and tests. These tests were performed according to the test procedure in the previous section The purpose of this test was to check whether the tourniquet sample could meet the requirements of use, i.e., whether the pressure data could exceed 300 mm Hg. In addition, the test results recorded the pressure at each turn of windlass (180 degrees) and the different pressure data caused by the different parts of the tourniquet on the Tournbutton – M. Performance tests to demonstrate the tourniquet tester was adequate for a range of tourniquet types is shown in Table 7. Then to demonstrate reproducibility of a distributed manufacturing of a tourniquet the Canadian parts sample manufactured in Poland was repeated four times. These results are shown in Table 8. As can be seen in Table 7, Table 8, first all tourniquets were able to apply the clinical maximum rates pressures without breaking. Table 7 also shows the placement of the buckle on the sensor has an impact on the measured pressure and it is recommended that the buckle is placed on the sensor directly to have reproducible values. There was, however, unsurprisingly a range of recommended turns needed to get this pressure from the different tourniquet designs. Lastly, as seen by Table 8, the distributedly manufactured tourniquets did indeed have variability. Thus, even the recommended number of turns changes. It is thus recommended that each distributedly manufactured tourniquet be tested and have the recommended number of turns be placed on the package or strapping material in permanent marker.

**Table 7:** Test results for multiple types of tourniquet designs.

**#**	**Tourniquet Type**	**Country**	**Date**	**Pressure (mmHg)**	**Number of Turns**	**Remarks**	**Break?**
**1**	*Canada 3D Parts*	Poland	April 2023	93	1	Plastic buckle of tourniquet on sensor	No
				390	3		No
				110	1	Fabric strip of tourniquet on sensor	No
				250	2		No
				305	2.5		No
**2**	*Glia Canada Sample*	Canada	January 2023	18	1	Fabric strip of tourniquet on sensor	No
				75	2		No
				163	3		No
				228	4		No
				333	5		No
				60	1	Plastic buckle of tourniquet on sensor	No
				169	2		No
				205	3		No
				396	4		No
**3**	*Glia Gaza Tourniquet*	Palestine	N/A	11	1	Fabric strip of tourniquet on sensor	No
				31	2		No
				80	3		No
				120	4		No
				170	5		No
				220	6		No
				202	6.5		No
				30	1	Plastic buckle of tourniquet on sensor	No
				30	2		No
				76	3		No
				180	4		No
				388	5		No
				310	5.5		No
**4**	*Milspect 5038 Nylon 001*	Open Works, Baltimore, MD USA	N/A	175	1	Fabric strip of tourniquet on sensor	No
				335	1.5		No
				140	1	Plastic buckle of tourniquet on sensor	No
				395	2		No
**5**	*IM Buckle and Back Plate*	Poland	April 2023	73	1	Fabric strip of tourniquet on sensor	No
				171	2		No
				202	2.5		No
				140	1	Plastic buckle of tourniquet on sensor	No
				380	2		No

**Table 8:** Test results for reproducibility test on Canada 3D Parts tourniquets.

**#**	**Tourniquet Type**	**Country**	**Date**	**Pressure (mmHg)**	**Number of Turns**	**Remarks**	**Break?**
**1**	*Canada 3D Parts*	Poland	April 2023	93	1	Plastic buckle of tourniquet on sensor	No
				390	3		No
				110	1	Fabric strip of tourniquet on sensor	No
				250	2		No
				305	2.5		No
**6**	*Canada 3D Parts*	Poland	April 2023	95	1	Fabric strip of tourniquet on sensor	No
				265	2		No
				150	3		No
				154	1	Plastic buckle of tourniquet on sensor	No
				411	2		No
**7**	*Canada 3D Parts*	Poland	April 2023	137	1	Fabric strip of tourniquet on sensor	No
				261	2		No
				319	2.5		No
				60	1	Plastic buckle of tourniquet on sensor	No
				260	2		No
				373	2.5		No
**8**	*Canada 3D Parts*	Poland	April 2023	103	1	Fabric strip of tourniquet on sensor	No
				250	2		No
				370	2.5		No
				190	1	Plastic buckle of tourniquet on sensor	No
				360	2		No

## Limitations and future works

After testing, the load cell used is stable and has good linear characteristics. The user operation is simple and straightforward. Readings are visible directly on the screen on top of the tester, with no additional operation or instrumentation is required. Due to the simple design and principle, the tester is also inexpensive to produce. This device can thus perform the needed function of testing tourniquets both commercial and donation, produced either on a manufacturing line or in a distributed fashion. The open-source tourniquet tester is much less expensive than all of the commercial offerings summarized in Table 1. Many of the testers shown in Table 1 can also be used for training and have features specifically for this (e.g., they look like limbs and have synthetic blood that is stopped when adequate pressure is applied. For the testing of tourniquets none of these features are necessary. Future work is needed to determine if the device described in this paper would also be adequate for training. Overall, the device met the design goals and is ready for laboratory testing at tourniquet manufacturing sites of any kind. There are, however, several areas of future work and ways the system can be improved. First, it should be pointed out that the open-source tourniquet tester is validated for research purposes and has not undergone medical device regulatory scrutiny that may be required in some jurisdictions. Second, there are some potential improvements that can be made on the device itself. The printing time, the plastic used, and the cost can all be further reduced by shrinking the size of the assembly. This can in part be done by making a dedicated open-source board for this application or coupling the output of the device to a relatively ubiquitous cell phone for obtaining the readings. Secondly, the snap-fit part can be damaged if it is repeatedly opened and closed roughly. Therefore, it is recommended to reduce the number of disassembles, but also future work can investigate the use of other materials, improving the toughness of the design.

**Fig. 15:**
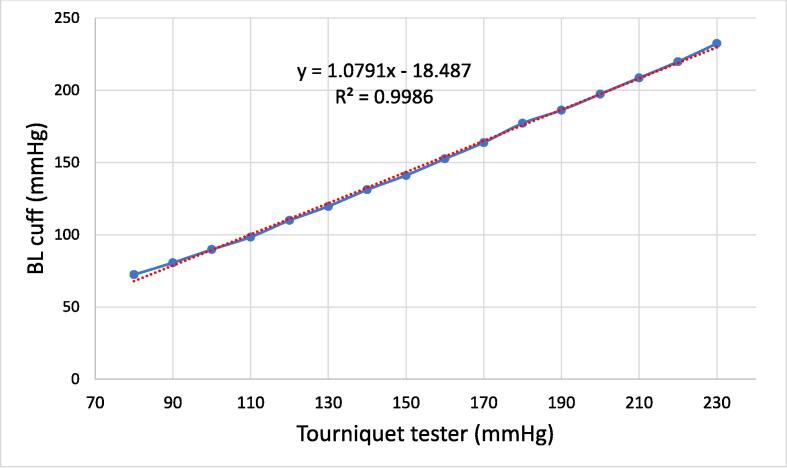
Validation tests, BL cuff vs tourniquet tester readings (p = 5).

There are some obvious limitations. Due to the housing being fixed, there is a maximum measuring limitation, but it is not the limitation of the tourniquet. It is because the pressure generated by the tourniquet comes from the deformation or compressing of the tourniquet. If the tourniquet cannot deform, then the force applied to the windlass will transfer to the strap itself, but not apply tothe cylinder. The TPU parts will help this situation, but the limitation still exists. Another limitation comes from the load cell: it has a measurement range, and if the force goes beyond its limit, the data will be inaccurate. The maximum force able to be measured on the system as designed is 200 N, which is the measurement range limit of the load cell (20 kg). It should be pointed out, however, that the value does not (and should not) go above 500 mmHg (approximately 154 N). After this amount of force is applied the patient can be damaged [82]. The tester should be deployed with this value labeled as a warning. Future work can also investigate the potential to make this a stand-alone (zero power) device by incorporating solar photovoltaic cells into the housing design. Other research groups associated with distributed tourniquet manufacturers can test additional types of tourniquets.

## Ethics statements

No human and animal subjects are used in this research.

## Funding

This work was supported by the Western Frugal Biomedical Innovation Strategic Grant, Glia, and the Thompson Endowment.

## Declaration of Competing Interest

The authors declare that they have no known competing financial interests or personal relationships that could have appeared to influence the work reported in this paper.

## Figures and Tables

**Table:** 

#	Tourniquet Type	Photographs	
1	*Canada 3-D Parts, assembled in Poland*		
2	*Glia Canada Sample*		
3	*Glia Gaza Tourniquet*		
4	*Milspect 5038 Nylon 001, Open Works, Baltimore, MD, USA*		
5	*IM Buckle and Back Plate*		

